# Syndrome de Guillain Barré et décompensation acidocétosique du diabète au cours de la grossesse: a propos d’un cas et revue de la littérature

**DOI:** 10.11604/pamj.2017.26.86.11091

**Published:** 2017-02-21

**Authors:** Lilia Affes, Mouna Elleuch, Fatma Mnif, Faten Hadj Kacem, Dhouha Ben Salah, Mouna Mnif, Nadia Charfi, Nabila Rekik, Mohamed Abid

**Affiliations:** 1 Service d’Endocrinologie Diabétologie, Hôpital Hedi Chaker de Sfax

**Keywords:** Acidocétose diabétique, Guillain Barré syndrome, grossesse, Diabetic ketoacidosis, Guillain Barré syndrome, pregnancy

## Abstract

Une femme enceinte âgée de 27 ans a été admise dans le service de réanimation pour une décompensation acidocétosique sévère spontanée inaugurale d'un diabète type 1. La patiente a été réanimée et insulinée avec une bonne évolution clinique et biologique. Au 4^ème^ jour, la patiente a présentée un tableau de polyradiculonévrite aigue d'installation brutale. Les examens complémentaires faites en urgences étaient négatives. Une cytoponction lombaire a montré une dissociation albuminocytologique. L'électromyogramme a confirmé le diagnostic de syndrome de Guillain Barré (SGB). La patiente a été mise sous veinoglobuline avec rééducation physique. Une amélioration spectaculaire des signes neurologiques a été notée. Concernant sa grossesse, la patiente a avorté au bout d'une semaine de diagnostic de SGB. L'association de SGB avec une décompensation cétosique est rare. En effet, quelques cas ont été rapportés dans la littérature. Cette association au cours d'une grossesse n'est jamais décrite d'où l'originalité de notre cas.

## Abstract (en)

A 27-year-old pregnant woman was admitted to the resuscitation department with severe spontaneous acute ketoacidosis as early symptom of type 1 diabetes. The patient underwent resuscitation and insulin treatment with good clinical and biological evolution. On day 4, the patient had polyradiculoneuritis characterised by acute onset. Additional emergency examinations were negative. Lumbar cytopunction showed albuminocytologic dissociation. Electromyogram confirmed the diagnosis of Guillain Barré syndrome (GBS). The patient was treated with veinoglobulin and underwent physical rehabilitation. A dramatic improvement in neurological signs was noted. With regard to pregnancy, the patient aborted a week after being diagnosed with GBS. The association of GBS with ketotic decompensation is rare. Indeed, a few cases have been reported in the literature. This association during pregnancy was never described, hence the originality of this case study.

## Introduction

Le syndrome de Guillain Barré est une polyradiculonévrite aigue de mécanisme auto-immun survenant suite à une infection virale ou bactérienne dans la plus part des cas. C'est une maladie rapidement progressive et habituellement réversible; cependant des conséquences fatales comme la défaillance respiratoire peuvent survenir. Son association avec le diabète type 1 notamment au cours de la grossesse est rarement décrite avec seulement cinq cas décrits dans la littérature. Nous rapportons le cas d'une décompensation acidocétosique inaugurale d'un diabète type 1 compliquée d'un syndrome de Guillain Barré chez une femme enceinte.

## Patient et observation

Il s'agit d'une patiente âgée de 27 ans, sans antécédents personnels particuliers et au antécédents familiaux chargés de maladies auto-immunes: diabète type 1 chez deux frères et thyroïdite de Hashimoto chez la mère. A 4 semaines d'aménorrhée soit un mois avant son hospitalisation, la patiente a présentée des signes cardinaux de diabète d'installation brutale avec un amaigrissement important. Devant l'accentuation des signes cardinaux et l'installation d'une altération d'état général, la patiente a été hospitalisée au service de réanimation pour une acidocétose diabétique. A l'examen, la patiente avait un état de conscience altéré avec un Glascow à 11/15 associé à une respiration ample et des signes cliniques de déshydratation extracellulaire. La tension artérielle systolique était à 100 mm Hg avec une fréquence respiratoire à 96/min. La glycémie initiale était à 5,4 g/l, l'acétonurie à 4 croix et la glycosurie à 4 croix. L'osmolarité était 304 mmol/l. Le gaz du sang a montré une acidose sévère avec PH à 7,1 et des réserves alcalines à 12 mmol/l. Elle a bénéficiée d'une réanimation avec hydratation et insulinothérapie par voie intraveineuse. L'évolution a été marquée par l'amélioration de l'état de conscience et d'hydratation, la disparition de l'acidose et l'amélioration de chiffres glycémiques ainsi que des troubles ioniques au bout de 3 jours. Au 4^ème^ jour de son hospitalisation, la patiente a présenté des paresthésies des 4 membres prédominant au niveau des pieds et des mains associées à une faiblesse musculaire d'installation rapide. Cette symptomatologie a évoluée rapidement vers une paralysie des 2 membres inférieurs associée à des dorsalgies et des crampes musculaires suivis par une béance sphinctérienne, dysarthrie, des troubles de la déglutition dans un contexte d'apyrexie se compliquant d'une défaillance respiratoire. Par ailleurs, des troubles neurovégétatives ont été notées avec une tachycardie à 120 battements par minute associé à une tension artérielle systolique à 100 mm Hg.

Le bilan biologique fait en urgence n'a pas montré des troubles ioniques ni de syndrome inflammatoire biologique. L'IRM cérébrale et médullaire, réalisée en urgence était normale. La ponction lombaire a montrée un liquide céphalo rachidien (LCR) clair avec culture négative. Toutefois, une dissociation albumino cytologique a été note avec une proteinorachie à 400 mg/dl et une cellularitée à 4/Ul. Devant le tableau clinique de polyradiculonévrite associé à la dissociation albumino cytologique, le syndrome de Guillain Barré a été suspecté. Le diagnostic a été confirmé par un électromyogramme (EMG) ayant montré un ralentissement de la conduction nerveuse. La patiente a bénéficiée d'une cure d'immunoglobuline à la dose de 0,4 g/kg/j en intraveineux durant 5 jours associée à une rééducation physique et une surveillance étroite. Une amélioration nette de signes neurologiques et neurovégétatifs a été objectivée. Une échographie obstétricale de surveillance, réalisée à 10 semaines d'aménorrhée soit deux semaines après la découverte de sa maladie, avait montrée une grossesse arrêtée. Par ailleurs, les troubles neurologiques ainsi que l'atteinte de système neurovégétative se sont améliorés au bout de 4 semaines. En parallèle, l'équilibre glycémique a nécessité 1 UI/kg/j d'insuline avec à l'enquête immunologique des anti GAD fortement positive faisant retenir le diagnostic de diabète type 1. Dans le cadre de l'enquête étiologique de SGB, l'interrogatoire n'a pas objectivé une infection ou un syndrome grippal dans les semaines précédentes. Les sérologies des germes atypiques, de Syphilis et les sérologies virales notamment celle de l'hépatite et de cytomégalovirus (CMV) et de Campylori Bacter Jejuni se sont révélées négatives.

## Discussion

Le syndrome de Guillain Barré est une polyradiculonévrite aigue due à une démyélinisation des nerfs périphériques secondaire à une réaction auto-immune. Trois quart des patients atteints de SGB ont souffert d'une infection virale ou bactérienne dans les jours ou les semaines précédentes le début des symptômes [1, 2]. Ces symptômes peuvent varier d'un rhume banal à des maux de gorges en passant par des troubles gastriques et intestinaux. Le CMV et le virus d'Ebstein Barr semblent être souvent le facteur déclenchant. Le Campylobacter Jejuni, responsable de certaines cas de gastroentérites constitue le facteur bactérien le plus souvent impliqué dans le déclenchement de SGB. Toutefois, les mécanismes par lesquels les virus ou les bactéries provoquent chez certaines personnes un syndrome de Guillain Barré ne sont pas encore élucidés [3, 4]. Les premiers symptômes comprennent des sensations anormales telles que des engourdissements, des picotements, des fourmillements se manifestant surtout dans les pieds et des mains associés à une faiblesse musculaire allant jusqu'à la paralysie. Ce dernier est d'évolution ascendante rapidement progressive et symétrique. Par ailleurs, des myalgies et des crampes musculaires peuvent se voire surtout au niveau du dos et des cuisses. Cette étape dure généralement une à trois semaines nécessitant l'hospitalisation dans un milieu de réanimation et se caractérise par un risque accru de fausses routes et d'une défaillance respiratoire, liée à la paralysie du diaphragme pouvant être fatale [5]. Ensuite, une phase en plateau succède et se caractérisant par une stabilisation des symptômes qui peut durer de quelques jours à quelques semaines. Cette phase peut aller jusqu'à l'apparition d'une quadriplégie associée à l'apparition de signes d'atteintes de système nerveux autonome : trouble du rythme cardiaque et variabilité tensionelle [6]. La gravité de cette étape est marquée par le risque thromboembolique lié à l'alitement prolongé et le risque infectieux respiratoire et urinaire. La récupération est observée dans 85% des cas [7]. Une évolution favorable est observée chez les sujets jeunes ayant un tableau d'installation brutale et d'intensité modérée et de durée courte.

Son traitement consiste à une hospitalisation immédiate dans un milieu de soin intensif. Le traitement doit être initié le plus rapidement possible, avant que les lésions des nerfs soient trop importantes. Deux traitements principaux, d'efficacité comparable, permettant de limiter le processus d'endommagement des nerfs. La plasmaphérèse consistant à remplacer le plasma du malade par du plasma sain nécessite plusieurs séances avec un risque accru de survenue d'effets indésirables. Le deuxième consiste à une injection d'immunoglobuline intraveineuse (Ig V) dont le rôle est de neutraliser l'auto anticorps. Le SGB peut survenir au cours de la grossesse avec une même incidence que celle de la population générale. Une cinquantaine de cas ont été publiés jusqu' à 2010 [8]. Toutefois seuls les cas sévères ont été rapportés [8–11]. Chan et al (2004)[4] ont rapporté dans une étude de 30 cas (de 1986 à 2002) une incidence annuelle de 0,86 pour 100 000 grossesses semblable à celle observée dans la population générale [12]. Le SGB peut apparaitre à n'importe quelle période de la grossesse [13]. Cependant l'incidence parait être plus élevée au cours du deuxième ou troisième trimestre, ou encore au premier mois du post partum [4]. Selon la série de chan et al, le syndrome de Guillan Barré se caractérisant par la gravité de l'atteinte motrice au cours de la grossesse et s'améliore rapidement en post partum [8]. D'autre part, SGB n'augmente pas le risque de fausse couche ni de mort fœtale. Il n'affecte pas non plus le développement du fœtus. Aucune étude n'a démontré l'augmentation de l'incidence d'avortement chez les femmes présentant un SGB au cours de la grossesse [13]. Seulement l'effet tératogène de CMV sur le fœtus est démontré. Par ailleurs, l'innocuité et l'efficacité de l'utilisation des Ig V durant la grossesse a été prouvée par plusieurs études [14]. Toutefois, un cas de SGB néonatale par transmission materno-foetale a été rapporté [15]. L'avortement chez notre patiente est expliqué probablement par l'acidocétose diabétique du au passage du corps cétoniques à travers la barrière placentaire occasionnant une hypoxie à l'origine d'une souffrance fœtale amenant à la mort fœtale in utero. L'association de SGB avec la décompensation cétosique du diabète a été rapportée seulement dans 5 cas dans la littérature Tableau 1 [7, 16–18]. Cependant, le diabète peut avoir un rôle dans l'évolution inhabituelle du SGB marquée par la persistance de l'atteinte neurovégétative [17]. La normalisation de la glycémie peut avoir un rôle favorable dans l'évolution de SGB [7] ce qui est le cas chez notre patiente ou chez qui l'évolution de SGB était habituelle. Dans la littérature, aucun lien étiopathogénique n'a été retrouvé entre la décompensation cétosique d'un diabète type 1 et le SGB [19]. Plusieurs auteurs ont suggéré que le diabète type 1 et le SGB peuvent partager un mécanisme auto-immun qui est déclenché très probablement par une infection fréquemment virale [17, 18]. Ce mécanisme est établi sur seulement des données épidémiologiques [19]. En effet aucun anticorps en commun n'a été trouvé jusqu' à maintenant [7]. D'autres auteurs suggèrent qu'il est possible que l'association entre SGB et diabète type 1 puisse être de type cause à effet et qu'une décompensation cétosique d'un diabète type 1 peut déclencher un SGB [7, 12, 20]. Certains auteurs suggèrent que le SGB doit être parmi les diagnostics différentiels d'une évolution neurologique inhabituelle au cours d'une décompensation d'un diabète [7]. Ce ci permet de ne pas méconnaitre un syndrome quiescent pouvant être fatal.

**Tableau 1:** Tableau résumant les cas publiés dans la littérature qui associent SGB et une décompensation acidocétosique d’un diabète

	Yusuke Kanemasa et al (2011) [7]	Noviello TB et al (2008) [16]	Fujiwara et al (2000) [17]	Rouan et et al (2000)(1) [18]	Rouan et et al (2000)(2)[18]
**Age**	64 ans	44 ans	37 ans	44 ans	25 ans
**Type de diabète**	Diabète type 2	Diabète type 2	Diabète type 2	Diabète type 2	Diabète type 1
**Ancienneté de diabète**	Diabète de primo découverte	Diabète de primo découverte	6 ans	Diabète de primo découverte	Diabète de primo découverte
**Cause de la décompensation**	Spontanée	Spontanée	Insulinopénie	Bronchite	Spontanée

## Conclusion

Le syndrome de Guillain Barré est une pathologie grave qui peut évoluer vers des formes sévères. Son association avec une décompensation cétosique du diabète est rarement décrite dans la littérature, et se révèle généralement par une forme atypique. Le SGB associé à une cétose inaugurale d'un diabète type 1 durant la grossesse est rapportée pour la première fois dans la littérature faisant évoquer des liens éthiopathogéniques probable entre les deux pathologies qui constituent jusqu'à ce jour un sujet de controverse.
